# Comparison of the low‐contrast detectability of two ultrasound systems using a grayscale phantom

**DOI:** 10.1120/jacmp.v17i6.6246

**Published:** 2016-11-08

**Authors:** Robert Lorentsson, Nasser Hosseini, Jan‐Olof Johansson, Wiebke Rosenberg, Benny Stenborg, Lars Gunnar Månsson, Magnus Båth

**Affiliations:** ^1^ Department of Medical Physics and Biomedical Engineering Sahlgrenska University Hospital Gothenburg Sweden; ^2^ Department of Radiation Physics Institute of Clinical Sciences at Sahlgrenska Academy University of Gothenburg Sweden

**Keywords:** ultrasound, gray scale, phantom, observation

## Abstract

The purpose of the present study was to use a commercially available grayscale phantom to compare two ultrasound systems regarding their ability to reproduce clinically relevant low‐contrast objects at different sizes and depths, taking into account human observer variability and other methodological issues related to observer performance studies. One high‐end and one general ultrasound scanner from the same manufacturer using the same probe were included. The study was intended to simulate the clinical situation where small low‐contrast objects are embedded in relatively homogeneous organs. Images containing 4 and 6.4 mm objects of four different contrasts were acquired from the grayscale phantom at different depths. Six observers participated in a 4‐alternative forced‐choice study based on 960 images. Case sample and human observer variabilities were taken into account using bootstrapping. At four of sixteen depth/size/contrast combinations, the visual performance of the high‐end scanner was significantly higher. Thus, it was possible to use a grayscale phantom to discriminate between the two evaluated ultrasound systems in terms of their ability to reproduce clinically relevant low‐contrast objects. However, the number of images and number of observers were larger than those usually used for constancy control.

PACS number(s): 87.57.C‐, 87.63.dh

## I. INTRODUCTION

The purpose of the medical ultrasound system is to reproduce the echogenicity of the patient as accurately as possible. There are many steps involved from the actual examination to the outcome of the same. The acoustic properties of the patient, the transmitter unit, how the beams are formed, the properties of the transducer, the receiver, how the signals and the image are processed, the quality of the display, and finally the ability of the user to drive the scanner and probe and interpret the displayed information all affect the probability for a correct diagnosis. One of the quantitative objectives in the B‐mode image is to detect and, if possible, identify the presence of limited‐size tissue masses (lesions) within or adjacent to a reference tissue.^(1)^ This objective is closely related to low‐contrast detectability^(2)^ which consequently is an important performance measure of an ultrasound system.

Quantifying the performance of medical imaging modalities can be done either by using human or model (mathematical) observers. Since model observers generally involve simple detection and discrimination tasks — in which a known target profile embedded in noise is discriminated from a known alternative profile — the development of models has typically considered diagnostic features in terms of signal and noise characteristics.^(3)^ The measurements from a model observer, well designed for the task, are quick, precise, and objective. This makes model observers suitable for control of, for example, low‐contrast detectability.^4^, ^5^ In quality control, detecting a potential change in a specific performance measure for a given system is often the primary objective. Assessing the impact of the change, which may be difficult based on the outcome of a model observer, may be a secondary objective.

It is tempting to use a model observer instead of human observers also to measure differences in performance between systems or to assess the impact of a change for a given system. However, when, for example, new techniques (like beamforming, image processing, etc.) are released in a new model, the correlation between the human and model observer, even if shown for previous existing conditions^6^, ^7^ has to be validated for this new condition. Thus, as long as the performance measure is intended to relate to a clinical use of the images produced by the system by humans, it may be an advantage to base the assessment of the performance measure on human observers, as the problems of validity then are reduced. Compared with other imaging modalities, this is of special importance in ultrasound imaging, as the degree of user reliance in producing a clinically relevant image is high.

Human observer studies are often based on receiver operating characteristics (ROC) analysis^(8)^ or multiple‐alternative forced‐choice (MAFC) experiments. These methods can be used for measurements of the ability of the human observer to detect a signal for a given image modality. The ROC method has long since been the gold standard for evaluation of performance with clinically acquired medical images and for comparisons of competing imaging methods.^(9)^ The observer is asked if a signal is present in a series of images and also how confident the observer is of the answer. Based on the responses to normal images (signal‐absent images) and abnormal images (signal‐present images), an ROC curve is determined. The ROC curve describes the relationship between the sensitivity and the specificity as the confidence level is altered. The area under the ROC curve (AUC) provides an objective measure of the performance of the observer and can be transformed to the signal‐to‐noise ratio (SNR) of the observer for the task (also expressed as the detectability index, d’).^(9)^ The MAFC method is an alternative to evaluate the observer's performance and can be used if the observer's decision criterion is of no importance.^(9)^ In MAFC studies, the observer is asked to select which of M locations is most probable of containing a signal, where one of the M locations actually contains the signal and the other M‐1 alternatives contain only noise. The proportion of correct answers in an MAFC study (PC) can be transformed to d’, linking the outcomes of ROC and MAFC studies.^(9)^

Intra‐ and interobserver variability is always present in studies involving human observers. The outcome of a study based on a small number of images and observers may therefore be of limited value. This is also of relevance when trying to assess low‐contrast detectability, regardless whether methods such as ROC and MAFC studies or subjective assessments are used. Always using human observers in control of the low‐contrast detectability may therefore be impractical, as described by Thilander‐Klang et al.^(4)^ in computed tomography and Tapiovaara and Sandborg^(5)^ in fluoroscopic imaging. The latter compared four methods for measuring low‐contrast detectability: subjective assessment was compared with two kinds of AFC experiments using human observers and the SNR as determined by a model observer. The conclusion was that, if the observers are not the same from time to time or if the observations are separated by a long time interval, subjective assessment would need impractically large numbers of observers for a precise evaluation. Also the AFC method was found to be impractical for routine purposes, such as constancy tests in quality assurance, since the time for such evaluation could require more than one hour observation time per observer, as well as several observers, for relevant precision.

Despite the reliability problems associated with human observer studies, such studies may nevertheless be needed when validity is essential. The highest validity is normally obtained if the images included are images of humans, as this results in a test situation most resembling the clinical situation. However, as patient anatomy varies much, acquiring an image material providing a fair comparison between systems may be difficult even if the patients are their own test and control. An additional difficulty of detection studies is that they rely on a known truth. If phantoms are used instead of patients, the experimenter has complete control over the ground truth and the acquisition conditions. However, for results based on phantom studies to be valid, the images produced by the phantom need to be relevant in terms of the appearance of the image background and the objects used to represent signals. Nevertheless, if the size, contrast, and depth of the objects in the phantom are clinically relevant, such studies may be of value in the comparison of different systems.

The purpose of the present study was to use a commercially available grayscale phantom to compare two ultrasound systems regarding their ability to reproduce clinically relevant low‐contrast objects at different sizes and depths, taking into account human observer variability and other methodological issues related to observer performance studies. The study was intended to simulate the clinical situation where small low‐contrast objects are embedded in relatively homogeneous organs.

## II. MATERIALS AND METHODS

### A. Phantom and image acquisition

**Figure 1:**
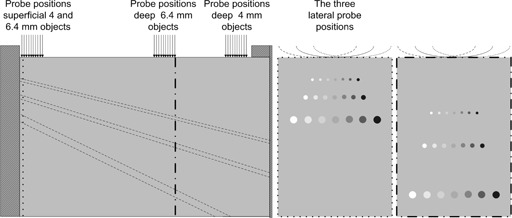
The left part shows the placement of the cylindrical objects as seen from the side of the phantom. To the right the placement of the objects when the phantom is scanned at the marked slices. The arrows show where the images of the objects were acquired for the three lateral positions of the probe.

As described above, the present work was restricted to simulate the situation where small low‐contrast objects (e.g., lesions) are embedded in relatively homogeneous organs in the abdomen (e.g., the liver), where speckle is the dominating image property affecting detection. The best way to perform a comparison of low‐contrast lesion detectability with real observers would be to choose a tissue mimicking phantom that makes it possible to acquire many statistically independent views of each target^10^, ^11^ from a phantom containing spherical objects of different sizes, contrasts and depths.^7^, ^12^, ^13^ None of the six investigated manufacturers of commercial test equipment (CIRS, Gammex, Blue Phantom, Kyoto Kagaku, ATS, and Dansk Fantomservice) has a phantom like this in their product catalog. The grayscale phantom CIRS 047 (Computerized Imaging Reference Systems, Norfolk, VA) fulfills all criteria, except that the objects are cylinders instead of spheres. This phantom was thus used in the present study. The phantom contains three different object sizes, 2.4, 4, and 6.4 mm, formed as cylinders (Fig. 1). Every size has seven different contrast levels: anechoic, ‐9dB,‐6dB,‐3dB,+3dB,+6dB, and +9dB. The depth possible for scanning varies for the different sizes: 1–6 cm for 2.4 mm, 2–9 cm for 4 mm, and 3–12 cm for 6.4 mm, according to the specifications. The maximum depth for the 2.4 mm objects is 6 cm and in abdominal examinations, the depth of the region of interest (ROI) is often deeper. Regarding the clinical relevance of lesion size, a retrospective clinical study of liver lesions^(2)^ has shown that the findings start between 4 and 5 mm. For these reasons, the 2.4 mm signals were excluded in the present study. The phantom was placed on a router table with ability to move the phantom in two directions with a precision of 0.1 mm. The probe was held by a clamp and in the same position while the phantom could move freely under the probe. Water was used as coupling medium. To get multiple reasonably independent samples of the objects regarding the speckle, inside and outside the objects, the phantom was moved both sideways and along the long side. For each object size the phantom was placed in three different locations sideways 15 mm from each other (three lateral positions) and 10 steps of 2 mm along the long side (three different angles between the vertical line from the center of the probe and the line from the center of the probe to the center of the object for each slice position) (see Fig. 1). In this way, 30 images of each object size were acquired at each of two depth regions (deep and superficial) of the phantom for both of the scanners (leading to a total of 240 images). The depths were 35–42 mm (superficial) and 90–98 mm (deep) for the 4 mm objects and 53–64 mm and 110–121 mm for the 6.4 mm objects. The images were saved as DICOM (Digital Imaging and Communications in Medicine) images and transferred to a personal computer.

### B. Scanners and settings

Two ultrasound systems were included in the present study, a high‐end GE Logiq9 (L9) and the more general GE Logiq P5 (LP5) (GE Healthcare, Milwaukee, WI). In order to isolate the capability of the scanner itself, the same probe was used for both scanners (curved array C4 standard probe (2–5 MHz)). The probe was tested and approved by the standards of Sonora FirstCall test system (Sonora Medical Systems, Inc., Longmont, CO). The default Abdomen settings, presented in Table 1, were used for the systems and the gain was adjusted to achieve a comfortable brightness on the monitor on the scanner. Time gain compensation was kept in central position. Depth was kept constant during image acquisition for each object size. No zooming was used. A single focus was set and positioned to be equal (or one step lower if the next step was above) to the actual object depth in each image. For each scanner, the same probe port was used for all image acquisitions.

**Table 1:** Abdomen setting for L9 and LP5

*Setting*	*Abdomen L9*	*Abdomen LP5*
Acoustic power output	100%	100%
Fundamental or harmonics	fundamental	fundamental
Smoothing	none	low
Compounding	low	none
Dynamic range	72	72
Speckle reduction imaging	3	0
Frequency	4 MHz	5 MHz
Grayscale map	D/0/0	C/0/0

### C. Observer study

**Figure 2:**
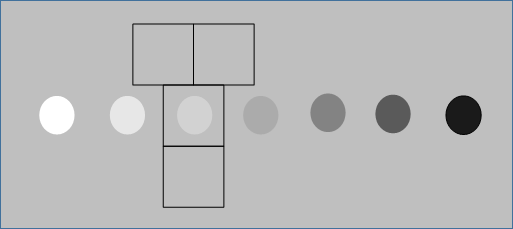
The principle of where the +3dB signal and the three background regions containing no signal were extracted.

Using MATLAB (MathWorks, Inc., Natick, MA), the acquired images were used to produce a dataset suitable for a 4‐alternative forced‐choice (4‐AFC) study. Around each signal, three background regions were extracted from the image (Fig. 2).

**Figure 3:**
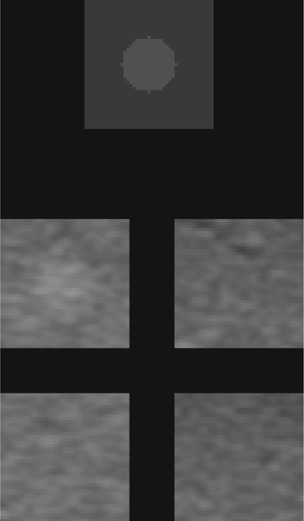
An example of the 4‐AFC test image. The object on top indicates the size, position, and contrast relative to the background to the observer.

The four regions (11 mm and 9 mm squares for the 6.4 mm and 4 mm signals, respectively) were used to produce 4‐AFC images. Each of the contrast levels +6,+3,+3, and +6dB were extracted from the same image for every combination of scanner, object size, and depth. The position of the signal image was randomized for each of the 4‐AFC images. As an aid, a reference square containing an object of the same size and contrast as the signal was shown on top of each image^10^, ^14^, ^15^ (Fig. 3).

Six observers participated in the 4‐AFC study, five medical engineers and one medical physicist. All observers had experience of ultrasound imaging and four of them regularly performed image quality control of medical imaging systems. The instruction to the observers was to determine which of the four squares contained the signal. No time limitation was set, the viewing distance was free, and the observers were free to alter zoom and window/level. Nearest‐neighbor pixel value interpolation was used and the default zoom level was twice as many pixels along one side as the original size. ViewDEX^16^, ^17^, ^18^ was used for presenting the images in a random order for each observer and recording the observers’ choices. The ambient lighting was kept at a low constant level, and the images were presented on a DICOM‐calibrated screen, EIZO Radiforce RX 320 (EIZO Corporation, Ishikawa, Japan). In total, each observer analyzed 960(2object sizes×4contrast levels×2depths×2scanners×30images) 4‐AFC images. The observers were not blinded to the design of the experiment, but they were blinded to the origin of the images in the experiment.

### D. Statistical analysis

For each combination of contrast, size, and depth, PC averaged over observers (n=6) and images (n=30) was determined as figure‐of‐merit (FOM). In order to correctly take all sources of uncertainty into account, bootstrapping was used to determine the uncertainty of the FOM. No assumptions about the underlying distribution are needed when using nonparametric bootstrapping.^(19)^ Bootstrapping is a resampling technique that can be used to estimate certain properties of a population from a single sample by generating multiple resamples (bootstrap samples) from the original sample. A bootstrap sample of the same size as the original sample is generated by sampling with replacement from the original sample. Each bootstrap sample constitutes an estimation of a real sample from the true distribution. Ten thousand (10,000) bootstrap samples were generated for each combination of contrast, size, and depth, where each sample consisted of 30 images sampled with replacement and six readers sampled with replacement to get a result that is generalizable to both the population of observers and the population of images. The proportion of correct responses was calculated for each bootstrap sample, resulting in a bootstrap distribution of PC. From the bootstrap distribution of PC, the nonparametric 95% confidence interval (CI) and the coefficient of variation (CV) of the FOM were determined. The percentiles 2.5 and 97.5 of the bootstrap distribution were used as the 95% CI and the CV was determined as ratio between the standard deviation of the bootstrap distribution of PC and the actual FOM. In two cases, the proportion of correct answers was 1 (FOM=1). Additionally, for some of the confidence intervals PC=1 was included. In these cases it is impossible to numerically transform PC to d’ since the value of d’ that corresponds to PC=1 is plus infinity. For this reason, only PC is presented in the results, as in the study of Miéville et al.^(20)^ and no attempt to convert the FOM to d’ was made. A similar bootstrap technique was used to determine the uncertainty of the difference in the FOM between the two scanners. The p‐values were calculated using a permutation test.^(19)^ Due to the many comparisons being made, a two‐sided p‐value <0.01 was considered indicating a statistically significant difference.

The variance for a single observer is given by the binomial distribution with the variance

$$s{\left(PC\right)}^{2}=PC(1-PC)/N$$

where *N* is the number of independent trials per data point (30 images in the present study). The CV for the single observer was determined based on this expression of the variance. To demonstrate the necessity of taking possible dependencies in the data into account in the statistical analysis, as a comparison the result of using the product of the number of images and the number of observers as N was calculated — as if every new image/observer combination was a unique trial (which was not the case since all observers viewed the same images). This result was compared with the CV determined from bootstrapping, as described above.

To put the obtained results between the scanners in perspective, comparisons between different contrast levels (±3dB vs. ±6dB, in total four comparisons) were made for the eight combinations of fixed scanner (n=2), size (n=2) and depth (n=2). Thus, this resulted in 32 comparisons where it was expected to obtain a significant difference in PC. Since the result was expected to be better for the ±6dB contrast levels, a one‐sided p‐value <0.01 was used to evaluate if the differences were significant. Additionally, comparisons where no difference in PC was expected were also performed: the positive and negative contrasts for 3 and 6 dB were compared to each other when scanner, size, and depth were fixed. This resulted in 16 comparisons and a two‐sided p‐value of 0.01 was set as a limit for significant difference. Finally, the observers’ results were compared to each other pairwise. In this comparison, all data from each observer were included and a two‐sided p‐value <0.01 was used to determine a significant difference in PC between each observer pair.

## III. RESULTS

The mean response time for all observers was 7 s per image. The proportion of correct answers for the 32 different combinations of contrast, size, depth, and scanner are shown in Table 2. For the 6.4 mm, ‐6dB superficial objects all observers answered correctly for both scanners. The lowest PC was 0.42 for 6.4 mm 3 dB at the deepest part of the phantom for L9. A statistically significant difference in PC between the two scanners was found for four of the 16 depth/size/contrast combinations. For these data points the high‐end scanner L9 achieved a higher PC than LP5.

**Figure 4:**
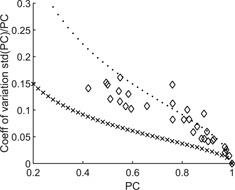
The coefficient of variation (CV) for different PC. Dots are the CV for single observers. Diamonds are the CV for bootstrapped cases and observers. X shows the level if the individual cases are divided by the square root of number of observers.

In Fig. 4 the CV determined for single observers and in different ways for the six observers in total for the different PCs is shown. The uncertainty for multiple observers was substantially underestimated if the analysis did not take the correlation between observers into account.

All 32 comparisons between the different contrast levels (±3dB vs. ±6dB) showed significant differences (p<0.01) when the scanner, size, and depth were fixed. For the 16 comparisons between the positive and negative contrast levels of the same strength, three comparisons showed significant differences when the scanner, size, and depth were fixed.

**Table 2:** PC (proportion of correct answers) for the six observers in the four‐alternative forced‐choice study. The percentiles 2.5 and 97.5 from the bootstrapped data were used as 95% confidence interval (CI)

*Contrast dB*	*Object Size (mm)*	*Depth (mm)*	*L9 PC (95% CI for PC)*	*LP5 PC (95% CI for PC)*	*p‐value*
‐6	4	35–42	0.97 (0.92–1.00)	0.89 (0.80–0.96)	<0.01
		90–98	0.76 (0.63–0.87)	0.88 (0.79–0.95)	0.08
	6.4	53–64	1.0 (1.0–1.0)	1.0 (1.0–1.0)	–
		110–121	0.86 (0.73–0.96)	0.91 (0.82–0.97)	0.29
‐3	4	35–42	0.59 (0.47–0.71)	0.55 (0.43–0.68)	0.42
		90–98	0.58 (0.42–0.73)	0.57 (0.46–0.69)	0.83
	6.4	53–64	0.84 (0.74–0.93)	0.81 (0.67–0.92)	0.31
		110–121	0.54 (0.39–0.69)	0.50 (0.34–0.64)	0.41
3	4	35–42	0.83 (0.68–0.94)	0.66 (0.51–0.78)	<0.01
		90–98	0.50 (0.36–0.66)	0.55 (0.37–0.72)	0.42
	6.4	53–64	0.89 (0.69–1.0)	0.76 (0.53–0.94)	<0.01
		110–121	0.42 (0.31–0.54)	0.51 (0.39–0.63)	0.14
6	4	35–42	0.98 (0.92–1.0)	0.90 (0.78–0.98)	<0.01
		90–98	0.76 (0.57–0.91)	0.82 (0.63–0.96)	0.25
	6.4	53–64	0.99 (0.95–1.0)	0.97 (0.9–1.0)	0.29
		110–121	0.93 (0.83–0.99)	0.92 (0.83–0.98)	0.86

Differences among the observers were seen. Observers 2–6 performed significantly better (p<0.01) than Observer 1, Observers 3–6 significantly better than Observer 2, and Observer 6 significantly better than Observer 3.

## IV. DISCUSSION

The purpose of the present study was to use a commercially available grayscale phantom to compare two ultrasound systems regarding their ability to reproduce clinically relevant low‐contrast objects at different sizes and depths, taking into account human observer variability and other methodological issues related to observer performance studies. The study was intended to simulate the clinical situation where small low‐contrast objects are embedded in relatively homogeneous organs. A 4‐AFC study was conducted in which human observers evaluated images of a grayscale phantom. The reason for choosing 4‐AFC instead of 2‐AFC was the better coefficient of variation^(10)^ even if 2‐AFC may be a more used figure of merit and it yields the AUC in an ROC study directly. A statistically significant difference between the image quality of the two included scanners was found for four of sixteen combinations of contrast level, object size, and depth, showing a higher performance for the high‐end scanner at shallower depths. The fact that a statistically significant difference between the two types of scanners in the present study (a general and a high‐end scanner) was found in only 25% of the detection tasks, despite six observers and 30 independent images per task, indicates that the difference between the two scanners for this type of tasks is relatively small. However, major factors affecting the relevance of using a grayscale phantom to evaluate low‐contrast detectability are the clinical validity of the results, depending on the detection task and the phantom used, and the study design. These issues will be discussed below.

Regarding the clinical relevance of the objects used, a retrospective clinical study of liver lesions^(2)^ has shown that the findings start between 4 and 5 mm. As mentioned before, partly for this reason the 2.4‐mm objects were excluded in the present study. Furthermore, a study investigating the characteristics of intra‐abdominal cystic masses showed that a variation in the number of internal echoes could be found in all the cystic masses examined.^(21)^ It is therefore an advantage to include a range of contrast levels when studying the ability of a system to reproduce low‐contrast details.

Regarding the clinical relevance of the background, the properties of different tissue‐mimicking materials in ultrasound phantoms have previously been studied by Browne et al.^(22)^ They found that the acoustic velocity in Zerdine (used in the CIRS 047) remained constant (±3 m/s) with increasing frequency but that the attenuation was nonlinear, which could affect penetration depth at higher frequencies. As long as the compared objects are far from the penetration depth, this effect would probably be minimal. The backscatter properties of the tissue‐mimicking material in the CIRS 047 are not mentioned in the specification from the manufacturer other than “scatter controlled independently from attenuation”. The backscatter coefficient is difficult to measure accurately and is therefore rarely reported in the literature.^(23)^ Nevertheless, the amount of scatter in the phantom results in images with a soft‐tissue‐like appearance. This makes this phantom suitable to simulate a clinical situation where the speckle dominates over the anatomical variations in a relatively homogenous background and the task is to detect lesions, although the absence of other anatomical signs that the examiner may take into account when searching for lesions results in a limited validity.

Regarding the phantom properties, to use objects formed as cylinders to evaluate the ability to detect spherical lesions has been questioned, since the effect of the slice thickness on the image is not taken in account.^7^, ^12^ Comparing the performance of different systems regarding contrast‐detail detectability could be misleading if the elevational resolution differs much between the systems at the actual depth. To avoid this problem, the best way to perform this kind of comparison with real observers would be to acquire a number of images, where the speckle is uncorrelated, with spherical low‐contrast objects from a phantom containing spherical lesions of different sizes, contrasts, and depths. As mentioned before, a commercial, serial‐produced phantom with these properties was not found. The spherical lesion phantom Gammex 408 LE (Gammex RMI, Middleton, WI) has 211 4 mm and 105 2 mm anechoic spheres at several depths. Using this phantom it would be possible to correctly take the partial volume effect of the elevational thickness into account, but the possibility of acquiring the necessary number of independent images would be much smaller than with the phantom used in the present study. Furthermore, the Gammex 408 LE phantom contains one contrast only. The Gammex 408 LE could thus be used in an observer study to show how deep a system reproduces anechoic 2 and 4 mm objects. However, below the dead zone and down to the deep limit the anechoic objects are probably too visible to be useful in an AFC study. This is the reason why ±6dB were the highest contrasts used in the present study. Regarding producing accurate images, another difference between using spherical and cylindrical objects is that phantoms containing spherical objects are more sensitive to the position of the probe. The beam has to align very accurately with the plane intersecting the center of the spheres. The position is important for the cylindrical objects as well, but an error in position affects what is being reproduced much more with the spheres. The presented method is a compromise between acquiring many independent images of the same objects of different contrasts and the bias of the partial volume effect when using cylinders instead of spheres, when using a commercially available ultrasound phantom.

Another issue is the angle between the vertical line from the center of the probe and the line from the center of the probe to the center of the object. Hall et al.^(2)^ show examples of decreased visibility when targets are positioned 22.5° off‐center. The presented method of acquiring images of the phantom using three different vertical placements of the probe results in a variety of angles. The largest angle was 42° while for 807 of the 960 extracted objects the angles were below 22.5°. The information in the periphery of the image is available for the examiner and it is reasonable to also include it in the result. A recently presented method for assessing imaging performance of medical ultrasound systems^(24)^ counts lesions in the whole image.

Regarding the study design, the images in a multiple‐alternative forced‐choice study should be statistically independent.^(10)^ The method used here produced three different angles when the phantom was moved in 15‐mm steps in the lateral direction and 10 different slices of the objects when the phantom was moved in 2‐mm steps in the elevation direction. Based on visual inspection, it was judged that the speckle changed enough when moving the phantom in the described directions to treat the images as independent, but no other analysis of the independence has been performed. Regarding the location of the object in the signal image, this should be exactly known by the observer in an AFC study. In the present study there were some difficulties in establishing the true location of the signal in certain images, hence an uncertainty was introduced for the observers. However, the possible misalignment was small enough for this problem to be deemed of limited value. Thus, it seems possible to use the investigated phantom to produce a statistically relevant image material valid for conducting observer performance studies. The regions containing no signal were extracted just above and below the signal regions, as shown in Fig. 2. The reason for this was that the geometry of the phantom made it difficult to collect them elsewhere since there was not enough space between or beside the objects. It would have been preferable to collect them at equal depths since the speckle size is affected by the depth and transmitted focus.^(25)^ However, a visual inspection could not reveal any significant differences in the appearance of the speckle, indicating that the effect of this was small.

A significant problem when comparing ultrasound systems is to choose what the settings should be. One single set of settings may be preferred to keep the amount of images at a reasonable level, even if this is not the way ultrasound is used in the clinical situation where the user instead often optimizes the settings depending on the task. There are mainly three ways to handle a single set of settings when comparing ultrasound systems. The first is to have standardized settings for the systems, which to some extent simplifies the comparison. However, some of the features may have to be suspended if those features are not available for all of the compared systems, leading to an unfair comparison and somewhat invalid results. The second way is to allow a representative of each manufacturer to optimize the system for the task. Although this probably makes the best use of the systems, the outcome becomes dependent on the expertise of the representatives and it may thus be difficult to remap the results for general use. The third way, and the one chosen for the present feasibility study, is to use the default setting for a certain and relevant examination. (In the present study general Abdomen was chosen, as this setting is of high relevance for the clinical task intended to be emulated using the phantom.) The reason for this approach was to compare the systems using the settings that the manufacturer finds appropriate for a given type of examination using a certain combination of probe and scanner. In the present study the L9 used some (low) crossbeam (spatial compounding) for the Abdomen setting while the LP5 did not. This may have contributed to better visibility at shallower depths for the L9. Regarding the frequency the default setting was used here as well, which in this case was different for the two compared systems even if the same multifrequency probe was used. Kofler et al.^(7)^ found “negligible dependence of detectability on the nominal frequency of the transducer”, which makes the default setting reasonable as the choice of transducer frequency when comparing different systems. Additionally, using a high‐frequency probe for maximizing the performance at shallow depths may lead to difficulties at deeper depths.

A large issue of observer performance studies is the statistical uncertainty. The use of nonparametric bootstrap as a plug‐in principle^(19)^ is an easy way to simulate the distribution that represents the requested situation and the percentiles in the simulated distribution can be used to determine the confidence interval. If the result should be valid only for the used cases (images) and the observers that participated, there is no variability and the truth is the proportion of correct answers. However, if the result should be valid for a population of cases (represented by the used case sample), the cases are placed in a case pool and drawn with replacement with a sample size equal to the original sample size using the answers for all observers for the drawn samples. This results in a distribution generalizable for the cases but only valid for the actual observers, a fixed‐observer situation. The observers are different in their ability to detect the signals and the use of several observers makes them representatives for a population of observers. To simulate this observer population, the observers are placed in an observer pool and drawn with replacement with a sample size equal to the number of observers who participated. By letting the answers from the sampled observers be from the actual cases the result is a fixed‐case situation, generalizable for the observers but only valid for the used cases. When combining these two situations by resampling both observers and cases, the variability in the simulated distribution contains case sample variability as well as intra‐ and inter‐observer variability, which is the situation used in the present study.

It may be surprising that a significant difference between the scanners was found in only four of sixteen comparisons even if the number of images and number of observers were relatively large. This indicates that it may be hard to detect differences when all the sources of variability are correctly taken into account, especially when the variability of the observers is introduced. When comparing performance, it may nevertheless be necessary to base the study on human observers, even if it is laborious, to achieve a high clinical validity of the results. For quality control, on the other hand, the present study indicates that other methods may be meritorious. However, the quality control may be intended to take the clinical effect of a detected change into account. For example, a malfunctioning probe element may be acceptable as long as it does not affect the clinical use of the probe. In such instances, it may be of value to employ methods with a high clinical validity also for quality control.

To put the results between the scanners in perspective, 32 comparisons were made where a significant difference in PC was expected, since all comparisons were made between higher and lower contrast levels and everything else was constant. All of the comparisons showed the expected difference. Thus, the method resulted in no false‐negatives (or type II errors) in these comparisons. When the positive and negative values of the same contrast levels were compared when everything else was constant, 3 out of 16 comparisons showed significant differences. Assuming that there should be no difference in detection between the positive and negative contrasts of the same magnitude, this means that the method resulted in three false‐positives (or type I errors). Two of them were the 6.4‐mm objects at signal levels + and −6dB at the ground depth for the two scanners. All observers got all answers right for the negative contrast, whereas one observer missed two and five answers for the positive contrast for the two scanners. When the PC values are so close to 1 as these are, some missed values have large influence on the difference. These points using the largest sizes of the positive and negative 6 dB objects turned out to be too easy to see to get desired differentiation for a 4‐AFC study. The third point that showed a difference was the 4 mm objects at ±3dB signal at ground depth for L9. Studying Table 2, the PC for the +3dB signal is unexpectedly high, which is the reason for the significant difference found. That type I or type II errors may be committed in studies is an unavoidable effect of hypothesis testing, and that a few type I errors assumingly have occurred here is not unexpected given the multitude of comparisons being made. In general, though, these results indicate that the method is robust and that it does not result in an unexpectedly high rate of type I or type II errors. That the method found four significant differences between the two scanners, and that it in all cases was the same scanner that performed better, should strengthen the confidence that the method may be used to discriminate between ultrasound scanners in terms of their ability to reproduce clinically relevant low‐contrast objects.

In the present study the purpose of using several observers was mainly to get a result that was representative for a general observer, but by using several observers a better statistical uncertainty was also expected. Figure 4 shows that the CV improved for most of the 32 data points for six observers compared to a single observer. The CV was however larger than the result obtained by dividing the standard deviation for one observer by the square root of the number of observers, which would be a reasonable method if all observations could be treated as independent. This indicates that the CV calculated this way is an underestimation of the real uncertainty. Furthermore, as shown in the results, there were significant differences in the performance of the observers, which also leads to an increased variability.

Finally, a preliminary analysis of some of the data in the present study has been presented by Lorentsson et al.^(26)^ That study only included the detection of the 4 mm object at the superficial depth, for which L9 was superior to LP5 for three of the four contrast levels. The present study showed that L9 was superior to LP5 for only one of the additional 12 depth/size/contrast combinations of the total 16 combinations. Thus, finding significant differences in low‐contrast detectability between ultrasound systems may be more difficult than the given impression from the 2015 Lorentsson study.

## V. CONCLUSIONS

The purpose of the present study was to use a commercially available grayscale phantom to compare two ultrasound systems regarding their ability to reproduce clinically relevant low‐contrast objects at different sizes and depths, taking into account human observer variability and other methodological issues related to observer performance studies. A four‐alternative forced‐choice experiment was conducted in which the detectability of details of different size, contrast and depth was compared between two ultrasound scanners from the same manufacturer. The study was intended to simulate the clinical situation where small low‐contrast objects are embedded in relatively homogeneous organs. A bootstrapping technique was used to take case‐sample and human‐observer variabilities into account. For four of sixteen combinations statistically significant differences were obtained, in favor of the high‐end machine. Thus, it was possible to use a grayscale phantom to discriminate between the two evaluated ultrasound systems in terms of their ability to reproduce clinically relevant low‐contrast objects. However, the number of images and number of observers were larger than those usually used for constancy control.

## COPYRIGHT

This work is licensed under a Creative Commons Attribution 3.0 Unported License.

## Supporting information

Supplementary Material

## References

[1] Hill CR , Bamber JC , Crawford DC , Lowe HJ , Webb S . What might echography learn from image science? Ultrasound Med Biol. 1991;17(6):559–75. 1962358 10.1016/0301-5629(91)90026-s

[2] Hall TJ , Insana MF , Harrison LA , Soller NM , Schlehr KJ . Ultrasound contrast‐detail analysis: a comparison of low‐contrast detectability among scanhead designs. Med Phys. 1995;22(7):1117–25. 7565387 10.1118/1.597505

[3] Abbey CK and Eckstein MP . Observer models as a surrogate to perception experiments. In: Samei E and Krupinski E, editors. The handbook of medical image perception and techniques. Cambridge UK: Cambridge University Press; 2010 p.240–50.

[4] Thilander‐Klang A , Ledenius K , Hansson J , Sund P , Båth M . Evaluation of subjective assessment of the low‐contrast visibility in constancy control of computed tomography. Radiat Prot Dosim. 2010;139(1–3):449–54. 10.1093/rpd/ncq069 20176732

[5] Tapiovaara MJ and Sandborg M . How should low‐contrast detail detectability be measured in fluoroscopy? Med Phys. 2004;31(9):2564–76. 15487739 10.1118/1.1779357

[6] Lopez H , Loew MH , Goodenough DJ . Objective analysis of ultrasound images by use of a computational observer. IEEE Trans Med Imaging. 1992;11(4):496–506. 18222891 10.1109/42.192685

[7] Kofler JM Jr , Lindstrom MJ , Kelcz F , Madsen EL . Association of automated and human observer lesion detecting ability using phantoms. Ultrasound Med Biol. 2005;31(3):351–59. 15749558 10.1016/j.ultrasmedbio.2004.12.003

[8] Swets JA , Pickett RM . Evaluation of diagnostic systems: methods from signal detection theory. New York: Academic Press; 1982.

[9] Burgess AE . The Rose model, revisited. J Opt Soc Am A Opt Image Sci Vis. 1999;16(3):633–46. 10069050 10.1364/josaa.16.000633

[10] Burgess AE . Comparison of receiver operating characteristic and forced choice observer performance measurement methods. Med Phys. 1995;22(5):643–55. 7643805 10.1118/1.597576

[11] Hall TJ , Insana MF , Soller NM , Harrison LA . Ultrasound contrast‐detail analysis: a preliminary study in human observer performance. Med Phys. 1993;20(1):117–27. 8455490 10.1118/1.597147

[12] Madsen EL , Zagzebski JA , Macdonald MC , Frank GR . Ultrasound focal lesion detectability phantoms. Med Phys. 1991;18(6):1171–80. 1753901 10.1118/1.596589

[13] Browne JE , Cannon LM , Fagan AJ . Comparison of in‐house development cylindrical and spherical anechoic target phantoms for performance testing of breast ultrasound scanners. Phys Med. 2014;30(6):718–24.

[14] Timberg P , Båth M , Andersson I , Mattsson S , Tingberg A , Ruschin M . In‐plane visibility of lesions using breast tomosynthesis and digital mammography. Med Phys. 2010;37(11):5618–26. 21158273 10.1118/1.3488899

[15] Timberg P , Båth M , Andersson I , Mattsson S , Tingberg A , Ruschin M . Visibility of microcalcification clusters and masses in breast tomosynthesis image volumes and digital mammography: a 4AFC human observer study. Med Phys. 2012;39(5):2431–37. 22559613 10.1118/1.3694105

[16] Börjesson S , Håkansson M , Båth M , et al. A software tool for increased efficiency in observer performance studies in radiology. Radiat Prot Dosim. 2005;114(1–3):45–52. 10.1093/rpd/nch550 15933080

[17] Håkansson M , Svensson S , Zachrisson S , Svalkvist A , Båth M , Månsson LG . ViewDEX: an efficient and easy‐to‐use software for observer performance studies. Radiat Prot Dosim. 2010;139(1–3):42–51. 10.1093/rpd/ncq057 20200105

[18] Svalkvist A , Svensson S , Håkansson M , Båth M , Månsson LG . ViewDEX: a status report. Radiat Prot Dosim. 2016;169(1–4):38–45. 10.1093/rpd/ncv543 26822421

[19] Efron B and Tibshirani RJ . An introduction to the bootstrap. Boca Raton: CRC Press LLC; 1993.

[20] Miéville FA , Gudinchet F , Brunelle F , Bochud FO , Verdun FR . Iterative reconstruction methods in two different MDCT scanners: physical metrics and 4‐alternative forced‐choice detectability experiments — a phantom approach. Phys Med. 2013;29(1):99–110. 22217444 10.1016/j.ejmp.2011.12.004

[21] Hill M and Sanders RC . Gray scale B scan characteristics of intra‐abdominal cystic masses. J Clin Ultrasound. 1978;6(4):217–22. 100514 10.1002/jcu.1870060404

[22] Browne JE , Ramnarine KV , Watson AJ , Hoskins PR . Assessment of the acoustic properties of common tissue‐mimicking test phantoms. Ultrasound Med Biol. 2003;29(7):1053–60. 12878252 10.1016/s0301-5629(03)00053-x

[23] Culjat MO , Goldenberg D , Tewari P , Singh RS . A review of tissue substitutes for ultrasound imaging. Ultrasound Med Biol. 2010;36(6):861–73. 20510184 10.1016/j.ultrasmedbio.2010.02.012

[24] Madsen EL , Song C , Frank GR . Low‐echo sphere phantoms and methods for assessing imaging performance of medical ultrasound scanners. Ultrasound Med Biol. 2014;40(7):1697–717. 24768482 10.1016/j.ultrasmedbio.2014.02.011 PMC4217281

[25] Oosterveld BJ , Thijssen JM , Verhoef WA . Texture of B‐mode echograms: 3‐D simulations and experiments of the effects of diffraction and scatterer density. Ultrason Imaging. 1985;7(2):142–60. 3909602 10.1177/016173468500700204

[26] Lorentsson R , Hosseini N , Johansson JO , et al. A 4‐AFC study comparing ultrasound machines using a grey‐scale phantom. Presented at the 16^th^ Nordic‐Baltic Conference on Biomedical Engineering. IFMBE Proc. 2015;48:82–85.

