# Supplementary material for: Comparison of the low‐contrast detectability of two ultrasound systems using a grayscale phantom
Source: J Appl Clin Med Phys. 2016 Nov 8;17(6):366–78. doi: 10.1120/jacmp.v17i6.6246 (PMC5690531; doi:10.1120/jacmp.v17i6.6246)
Supplement: Supplementary file 1 — Supplementary Material [file ACM2-17-366-s001.pdf]

A 4-AFC Study Comparing Ultrasound Machines Using a Greyscale Phantom

R. Lorentsson[1,2], N. Hosseini[1], J.-O. Johansson[1], W. Rosenberg[1], B. Stenborg[1], L.G. Månsson[1,2], and M. Båth[1,2]

[1] Department of Medical Physics and Biomedical Engineering, Sahlgrenska University Hospital, Gothenburg, Sweden
[2] Department of Radiation Physics, Institute of Clinical Sciences, Sahlgrenska Academy, University of Gothenburg, Sweden

Abstract— **Two ultrasound machines, one high-end and one ordinary, were compared in terms of their ability to reproduce low-contrast objects using the same probe. Images containing 4 mm objects of four different contrasts were collected from a greyscale phantom at 35-42 mm depth. Six observers participated in a 4-alternative forced choice study based on 120 images. At this certain depth and object size the proportion of correct responses was higher (statistically significant) for the high-end machine at three of four contrast levels, indicating the possibility to discriminate between ultrasound machines using a limited number of images of a greyscale phantom. However, the number of images and number of observers needed are larger than usually used for constancy control.**

Keywords— **Ultrasound, phantom observation, detection, 4-AFC.**

I. Introduction

Ultrasound is a common medical diagnostic technique in modern healthcare. The ultrasound machine park in a hospital consists of a wide range of ultrasound systems. There are all from low end ultrasound systems to expensive high end with several probes and specially designed software for certain examinations. This, together with the fact that some of the machines are in the end of their life cycle and some in the beginning typically results in a large variety of ultrasound equipment in a hospital. The brightness mode (B-mode) scanning produces an image of the tissue where each echo is presented as a bright dot, the stronger echo, the brighter dot. It is not obvious how the quality of the B-mode image can be quantified in an objective and clinically relevant way. The reason for wanting to quantify the image quality is both to see if a machine degrades over time and be able to compare different machines. The B-mode image has three quantitative objectives [1]

- To visualize anatomy on as fine scale as possible
- To detect and, if possible identify, the presence of limited tissue size masses (lesions) within or adjacent to a reference tissue
- To detect, observe or measure movement of a structure in time.

The present paper focuses on the second of the three, i.e. the possibility to detect low contrast objects of different contrast. The purpose of the study was to compare two different ultrasound machines from the same manufacturer, one high end and one ordinary, in terms of their ability to reproduce low contrast and taking into account human observer variability.

II. Method

A. Machines and settings

The two ultrasound machines evaluated were a high-end GE Logiq9 (GE Healthcare, Milwaukee, USA) purchased 2006 (L9), and the more ordinary GE Logiq P5 (LP5), purchased 2007. In order to isolate the capability of the machine itself, the same probe was used for both machines (curved array C4 probe, (2-5 MHz)). The Abdomen-setting was used for the machines and only the gain, depth and focus were adjusted for best viewable image. The L9 used 4 MHz and the LP5 5 MHz for the Abdomen-setting. A single focus was set and positioned for the actual object depth in each image.

© Springer International Publishing Switzerland 2015

H. Mindedal and M. Persson (eds.), *16th Nordic-Baltic Conference on Biomedical Engineering*,
IFMBE Proceedings 48, DOI: 10.1007/978-3-319-12967-9_22

B. Phantom and Image Acquisition

To evaluate objects of different contrast, the greyscale phantom CIRS 047 (Computerized Imaging Reference Systems, Incorporated, Norfolk, USA), was used. The phantom contains three different object sizes, 2.4, 4 and 6.4 mm, formed as cylinders. Every size has seven different contrast levels, anechoic, -9 dB, -6 dB, -3 dB, + 3 dB, +6 dB and +9 dB. The depth varies for the different sizes: 1-6 cm for 2.4 mm, 2- 9 cm for 4 mm and 3-12 cm for 6.4 mm. In the present study, only the 4 mm objects were used. The phantom was placed on a router table with ability to move the phantom in two directions with a precision of 0.1 mm. The probe was held by a clamp and in the same position while the phantom could move freely under the probe. Water was used as coupling medium. To get independent images of the objects according to the surrounding speckle the phantom was moved both sideways and along the long side. For each signal the phantom was placed in three different locations sideways 15 mm from each other and ten steps of 2 mm along the long side (three different angles for each slice position). In this way 30 images of the 4 mm objects for each of the contrast levels -6, -3, +3 and +6 dB were collected at 35-42 mm depth. The images were saved as DICOM images and transferred to a personal computer.

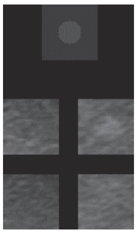

Fig. 1 An example of the 4AFC test object. The object on top indicates to the observer the size, position and contrast relative to the background.

C. Observer study

Using MATLAB R2013b (The Mathworks, Inc., Massachusetts, USA), the acquired images were used to produce a data set suitable for a 4-alternative forced choice (4-AFC) study. Around each signal, three background regions were extracted from the image. The four regions (1 signal region+3 background regions) were used to produce 4-AFC images, where the position of the signal image was randomized. As an aid, a reference square containing an object of the same size and contrast as the signal was shown on top of each image (Fig. 1). Six observers participated in the 4- 4-AFC study, five medical engineers and one medical physicist. The instruction to the observers was to determine which of the four squares that contained the signal. No time limitation was set, and the observers were free to alter zoom and window/level. ViewDEX [2, 3] was used for presenting the images in random order for each observer and recording the observers' choices. The ambient lightning was kept at a low constant level, and the images were presented on a DICOM-calibrated screen, EIZO Radiforce RX 320 (EIZO Corporation, Ishikawa, Japan). In total, each observer analyzed 120 images.

D. Statistical analysis

The proportion of correct responses (P) and the difference of P, P_{dif} between the two machines was calculated.

Bootstrap simulation was used to estimate the uncertainty. P and P_{dif} was bootstrapped 10 000 times for random combinations of observers and images for each contrast level. The percentiles 2.5 and 97.5 from the simulated data were used as 95% confidence interval (CI).

III. RESULT

Table 1 The average performance (proportion of correct responses, P) for the 6 observers in the 4-AFC study.

Contrast	L9 P (95% CI for P)	LP5 P (95% CI for P)
-6 dB	0.97 (0.92 - 1.00)	0.89 (0.80 - 0.96)
-3 dB	0.59 (0.47 - 0.71)	0.55 (0.43 - 0.68)
3dB	0.83 (0.68 - 0.94)	0.66 (0.51 - 0.78)
6dB	0.98 (0.92 - 1.0)	0.90 (0.78 - 0.98)

The result of the 4-AFC study for the two machines are presented in Table 1. At all contrast levels, the L9 had a higher value of P although the uncertainty was relatively large compared to the differences. Nevertheless, the difference was statistically significant at all contrast levels except – 3 dB (Fig 2).

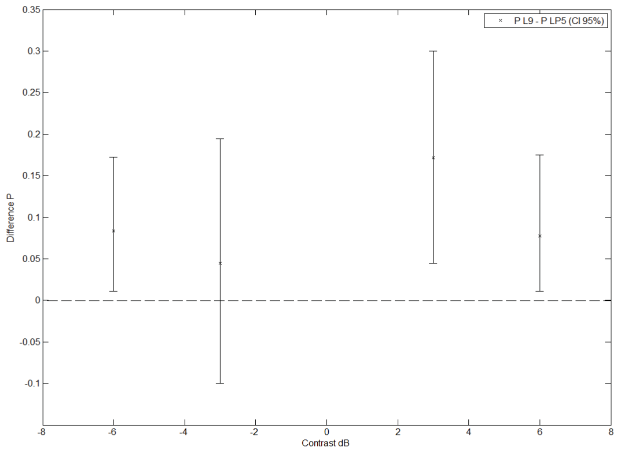

Fig. 2 The difference in P between L9 and LP5. The error bars represent 95% CI.

IV. Discussion

In the present study the ability of ultrasound machines to reproduce low-contrast object has been investigated. A 4-AFC study was conducted in which human observers evaluate images of a grayscale phantom. A statistically significant difference between the two included machines was found for three of four contrast levels, indicating a higher performance for the high-end machine in this task. The clinical relevance of this result depends on e.g. the validity of the detection task, how the phantom is constructed and the study design. These issues will be discussed below.

A. Images

The images in a multiple alternative forced choice should be statistically independent [4]. The method used here, produced 3 different angles from the object to the center of the transducer when the phantom was moved 15 mm in the lateral direction, and 10 different slices of the objects when the phantom was moved 2 mm in the elevation direction. Based on visual inspection, it was judged that the speckle changed enough when moving the phantom in the described directions to treat the images as independent, but no other analysis of the independence has been performed.

Regarding the location of the object in the signal image, this should be exactly known by the observer in an AFC study. In the present study there were some difficulties in establishing the true location of the signal in certain images, hence an uncertainty was introduced for the observers. However, the possible misalignment was small enough for this problem to be deemed of limited value.

B. Phantom properties

To use objects formed as cylinders to evaluate the ability to detect spherical lesions have been questioned [5, 6], since the effect of the slice thickness on the image is not taken in account. To avoid this problem the best way to perform this kind of comparison with real observers would probably be to collect a number of speckle-independent images with spherical low contrast objects from a phantom containing spherical lesions of different sizes, contrasts and depths. To our knowledge, none of the phantoms from the large manufacturers has a phantom like this in their product catalog. Either the spheres are just in one contrast (anechoic), or else it contains just one sphere of each contrast and size at most 3 depths, which makes it difficult to collect enough independent samples.

Another difference between using spherical objects and cylindrical is that phantoms containing spherical objects are more sensitive to the position of the probe in order to produce accurate images. The center of the beam has to align with the plane of the spheres in two directions to align with the center of the spheres. The position is important for the cylindrical objects as well but an error in the position affects what is being reproduced much more with the spheres. The presented method is a compromise between collecting many independent images of the same objects and the bias of the partial volume effect when using cylinders instead of spheres, when using a commercial available ultrasound phantom.

Regarding the clinical relevance of lesion size, a retrospective clinical study of liver lesions [7] has shown that the findings start between 4 and 5 mm. For this reason, the 2.4-mm signals were excluded in the present study. Furthermore, the visibility of the 6.4-mm signal was too high for relevant inclusion in a 4-AFC study. The 4-mm lesions included both had suitable visibility and a clinically relevant size. A study investigating the characteristics of intra-abdominal cystic masses showed that a variation in the number of internal echoes could be found in all the cystic masses examined [8]. It is therefore an advantage to include a range of contrast levels when studying the ability of a machine to reproduce low-contrast details.

The properties of different tissue mimicking materials in ultrasound phantoms have been studied [9]. The acoustic velocity in Zerdine™ (used in CIRS 047), remained constant (+- 3 m/s) but the attenuation were found to be nonlinear with increasing frequency, which could affect penetration depth at higher frequencies. As long as the compared objects are far from the penetration depth this effect would probably be minimal. The backscatter properties of the

tissue mimicking material in the CIRS 047 is not mentioned in the specification from the manufacturer other than “scatter controlled independently from attenuation”. The backscatter coefficient is difficult to measure accurately and therefore is rarely reported in the literature.[10] Nevertheless, the amount of scatter in the phantom results in images with a soft tissue-like appearance. The absence of other anatomical signs that the examiner take into account when searching for lesions makes a phantom study less valid, but an advantage is that it is easier to perform since the observers need no medical background, and the images are easy to make in large amounts. Also, the ability of machine to reproduce low contrast objects is an important property.

C. Uncertainty

The precision of an observer in a 4-AFC test depends on the number of observations and the probability of a correct answer. The relative standard deviation (s (d′)/d’) for one observer, P = 0.8 and 30 images is 0.18 [4]. By dividing by the RMS of number of observers [11] the relative standard error for present study is $\frac{0.18}{\sqrt{6}} = 0.073$. This simple estimation of the uncertainty of the results agrees reasonably well with the size of the confidence interval determined using bootstrapping, although the latter method more accurately takes into account all existent sources of variability.

V. CONCLUSION

The results of the present study indicate that even if inter-observer variability is correctly taken into account it is possible to discriminate between ultrasound machines, in terms of their ability to reproduce low-contrast details, using a limited number of images of a greyscale phantom. However, the number of images and number of observers needed are larger than usually used for constancy control.

REFERENCES

1. Hill C R, Bamber J C, Crawford D C et al. (1991) What might echography learn from image science? Ultrasound Med Biol 17:559-575
2. Borjesson S, Hakansson M, Bath M et al. (2005) A software tool for increased efficiency in observer performance studies in radiology Radiat Prot Dosimetry 114:45-52
3. Hakansson M, Svensson S, Zachrisson S et al. (2010) VIEWDEX: an efficient and easy-to-use software for observer performance studies Radiat Prot Dosimetry 139:42-51
4. Burgess A E (1995) Comparison of receiver operating characteristic and forced choice observer performance measurement methods Medical Physics 22:643-655
5. Kofler J M, Jr., Lindstrom M J, Kelcz F et al. (2005) Association of automated and human observer lesion detecting ability using phantoms Ultrasound Med Biol 31:351-359
6. Madsen E L (1991) Ultrasound focal lesion detectability phantoms Medical Physics 18:1171
7. Hall T J (1995) Ultrasound contrast-detail analysis: A comparison of low-contrast detectability among scanhead designs Medical Physics 22:1117
8. Hill M, and Sanders R C (1978) Gray scale B scan characteristics of intra-abdominal cystic masses Journal of Clinical Ultrasound 6:217-222
9. Browne J E, Ramnarine K V, Watson A J et al. (2003) Assessment of the acoustic properties of common tissue-mimicking test phantoms Ultrasound in Medicine & Biology 29:1053-1060
10. Culjat M O, Goldenberg D, Tewari P et al. (2010) A review of tissue substitutes for ultrasound imaging Ultrasound Med Biol 36:861-873
11. Tapiovaara M J, and Sandborg M (2004) How should low-contrast detail detectability be measured in fluoroscopy? Medical Physics 31:2564

Author: Robert Lorentsson
Institute: Sahlgrenska University Hospital
Street: Vita stråket 12
City: Gothenburg
Country: Sweden
Email: robert.lorentsson@vgregion.se
